# Negotiating policy in practice: child and family health nurses’ approach to the process of postnatal psychosocial assessment

**DOI:** 10.1186/1472-6963-13-133

**Published:** 2013-04-08

**Authors:** Mellanie Rollans, Virginia Schmied, Lynn Kemp, Tanya Meade

**Affiliations:** 1 School of Nursing and Midwifery, University of Western Sydney, Sydney, NSW, Australia; 2 Centre for Health Equity Training Research Evaluation (CHETRE), Centre for Primary Health Care and Equity, University of NSW, Sydney, 2052, Australia; 3 School of Social Sciences and Psychology, University of Western Sydney, Sydney, NSW, Australia

**Keywords:** Psychosocial assessment, Depression screening, Child and family health nurses, Perinatal mental health, Postnatal depression, Domestic violence screening, Home visiting

## Abstract

**Background:**

There is growing recognition internationally of the need to identify women with risk factors for poor perinatal mental health in pregnancy and following birth. In the state of New South Wales, Australia the Supporting Families Early policy provides a framework of assessment and support for women and families and includes routine psychosocial assessment and depression screening. This study investigated the approach taken by Child and Family Health Nurses (CFHNs) following birth to assessment and screening as recommended by state policy. This was a qualitative ethnographic study that included 83 CFHN and 20 women. Observations occurred with thirteen nurses; with 20 women, in the home or the clinic environment. An additional 70 nurses participated in discussion groups. An observational tool (4D&4R) and field notes were used to record observations and analysed descriptively using frequencies. Field notes, interview data and discussion group transcripts were analysed thematically.

**Methods:**

This was a qualitative ethnographic study that included 83 CFHN and 20 women. Observations occurred with thirteen nurses; with 20 women, in the home or the clinic environment. An additional 70 nurses participated in discussion groups. An observational tool (4D&4R) and field notes were used to record observations and analysed descriptively using frequencies. Field notes, interview data and discussion group transcripts were analysed thematically.

**Results:**

CFHNs demonstrated a range of approaches to assessment and screening. Psychosocial assessment was conducted in 50% (10 out of the 20) of the interactions observed; however, all the women were screened using the Edinburgh Depression Scale. Four major themes that represent the approach taken to the assessment process were identified: ‘Engagement: getting that first bit right’, ‘Doing some paperwork’, ‘Creating comfort’ and ‘Psychosocial assessment: doing it another way’. Nurses utilised other skills such as observing the women interacting with their baby, taking note of non verbal communication and using intuition to develop a clinical decision.

**Conclusion:**

Overall, nurses’ took a sensitive and caring approach to assessment and screening, however, there were differences in interpretations of the policy recommendations across the two sites. Nurses adopt a flexible, relationship-based approach to the assessment process; however, they experience tension when required to incorporate structured psychosocial assessment processes. To undertake assessment and screening effectively, CFHNs require ongoing support, training and supervision to maintain this sensitive and emotionally challenging work.

## Background

Psychosocial issues during pregnancy and early parenting are common and may have lasting effects with poorer outcomes for women and their families [1-3]. Prevalence of depressive symptoms or a diagnosis of depression ranges from 8 to 15% in the first year following birth [4] highlighting the need to identify and address psychosocial issues early [5]. The importance of early intervention has led to initiatives both in Australia and internationally to conduct routine assessment and screening of pregnant and postpartum women in order to identify those who may be at risk of adverse mental health outcomes.

All Australian states and territories provide universal child and family health nursing services free of charge to all children from birth to 5 and their families. In some instances CFHN will make contact with families in the antenatal period, although this is less common [6]. While the policy platform and schedule of services vary across states and territories, all offer routine monitoring of child development, health promotion activities focused on both children and families and the provision of support for early parenting and families [7]. Women are referred from the maternity service to the child and family health nursing service by midwives but as these services are generally not under the same management structure, there is little direct communication between CFHNs and midwives [8]. Levels of continuity of service and care provider vary greatly at both a state and local level. In all states and territories child family health nursing services are provided by registered nurses with specialist qualifications in child and family health nursing. Known in most Australian jurisdictions as child and family health nurses (CFHNs), they are similar to health visitors in the UK [9], child health nurses in Sweden [10] and plunkett nurses in New Zealand [11].

In the Australian state of New South Wales (NSW) the Supporting Families Early (SFE) policy and the Safe Start guidelines [12] formalised long standing practice that had been operating under draft guidelines since 2001 (IPC). This policy and guidelines outline the services to be provided to new parents to support child health and development in the postnatal period. A key recommended part of that role is assessment and screening for psychosocial risk, both antenatally and postnatally. This assessment is undertaken by midwives at the first antenatal appointment (around 12 to 14 weeks gestation) and by CFHN within the first six to eight weeks after birth. The policy recommends a structured assessment and screening process that incorporates a specific set of questions (see Table 1) and the use of the Edinburgh Postnatal Depression Scale (EPDS) [13], both before and after birth. The policy, however, also suggests that CFHNs take a flexible, partnership based approach to practice [12]. The tension between taking a structured approach to assessment and screening versus working in a flexible way has been raised by some commentators [9,14-16] who argue it is more appropriate to engage parents in a discussion of their needs.

**Table 1:** Psychosocial assessment and questions

**Variables (Risk factors)**	**Suggested format for psychosocial assessment questions**
I. Lack of support	1. Will you be able to get practical support with your baby?
	2. Do you have someone you are able to talk to about your feelings or worries?
II. Recent major stressors in the last 12 months.	3. Have you had any major stressors, changes or losses recently (i.e., in the last 12 months) such as, financial problems, someone close to you dying, or any other serious worries?
III. Low self-esteem (including lack of self-confidence, high anxiety and perfectionist traits)	4. Generally, do you consider yourself a confident person?
	5. Does it worry you a lot if things get messy or out of place?
IV. History of anxiety, depression or other mental health problems	6a. Have you ever felt anxious, miserable, worried or depressed for more than a couple of weeks?
	6b. If so, did it seriously interfere with your work and your relationships with friends and family?
	7. Are you currently receiving, or have you in the past received treatment for any emotional problems?
V. Couple’s Relationship Problems or Dysfunction (if applicable)	8. How would you describe your relationship with your partner?
	9. a) Antenatal: What do you think your relationship will be like after the birth?
	OR
	b) Postnatal (in Community Health Setting): Has your relationship changed since having the baby?
VI. Adverse childhood experiences	10. Now that you are having a child of your own, you may think more about your own childhood and what it was like. As a child were you hurt or abused in any way (physically, emotionally, sexually)?
VII. Domestic violence (DV) Questions must be asked only when the woman can be interviewed away from partner or family member over the age of 3 years. Staff must undergo training in screening for domestic violence before administering questions	11. Within the last year have you been hit, slapped, or hurt in other ways by your partner or ex-partner?
	12. Are you frightened of your partner or ex-partner? (If the response to questions 11 and 12 is “No” then offer the DV information card and omit questions 13–18)
	13. Are you safe here at home?/to go home when you leave here?
	14. Has your child/children been hurt or witnessed violence?
	15. Who is/are your children with now?
	16. Are they safe?
	17. Are you worried about your child/children’s safety?
	18. Would you like assistance with this?
Opportunity to disclose further	19. Are there any other issues or worries you would like to mention?

[11].

These initiatives, whilst important in the detection of potential risk factors, place greater emphasis on midwives and CFHNs as the frontline clinicians, to increase their knowledge of social and emotional risk factors for poor mental health during pregnancy and after birth [17]. The dynamic interaction between the woman and the CFHN during assessment of social and emotional needs is still poorly understood and often unrecognised in practice [15,18]. Few studies have investigated the process of psychosocial assessment and screening in the postnatal period.

In this current study, the authors have examined the process and impact of psychosocial assessment and screening on both women and the midwife/CFHN conducting the assessment at two points in time, in pregnancy and after birth, as outlined in the NSW policy recommendation [12]. The findings of the observations of midwife-woman interaction during assessment and screening in pregnancy are presented in Rollans et al. [19]. This paper examines the approach (actions and interactions) that CFHNs take to conduct this psychosocial assessment and screening in the early postnatal period. The process of assessment and CFHNs role is examined in the context of the above debates and policy that requires both the use of structured tools and assessments but also a flexible, partnership approach to working with families. The study explored how the nurses negotiate and make sense of these two potentially contradictory approaches in practice.

## Methods

This is an ethnographic study that was conducted in NSW, Australia between February 2011 and October 2011. Data was gathered from observations of CFHN-client interactions, brief interviews with the nurses following the clinical encounters and from five discussion groups conducted with CFHNs from the participating sites. Ethics approval for the study was obtained from the Human Research Ethics Committee in the two local health districts where the study was conducted and from the University of Western Sydney. The qualitative component of this manuscript adheres to the qualitative research review guidelines (RATS).

### Setting

The study was conducted in two local health districts in NSW. These sites were selected because the assessment and depression screening processes had been in place in the antenatal period for over five years; however, implementation of assessment following birth was more recent in both sites. Both sites underwent updates their processes in order to align with the new SFE policy. The two sites differed with regard to the timing of depression screening and psychosocial assessment. At site A, CFHNs ask women to complete the EPDS at the home visit that occurs within two to four weeks after discharge from hospital; whilst at site B, CFHNs screen with the EPDS when the woman visits the clinic six weeks after birth.

### Participants and recruitment

A total of 83 CFHNs and 20 women agreed to participate in the study. Of the 83 CFHNs, 13 were observed during their interaction with the 20 postnatal women and 70 additional nurses participated in discussion groups. Both the CFHNs and the women were recruited prior to the birth. Women were recruited in the antenatal clinic. In order to observe the same group of women interacting with CFHNs after birth, CFHNs were informed about and recruited to participate in the study through a series of in-service sessions conducted by the researchers at each site. The women participants observed after birth had also been observed in the antenatal booking visit. Women were excluded from the study if they were under 18 years old or required an interpreter. After birth, women participating in this study were linked to a consenting CFHN, who was to conduct the home visit or the six week clinic visit, at which time the first author (MR) was present to observe the interaction.

All CFHNs working in these two sites were also invited to participate in a discussion group. Information about the discussion group was presented at the in-service sessions and consent was obtained at the start of the group.

The professional experience of the participating CFHNs ranged from one year to over 20 years. The average age of the CFHN participants was 51 years, ranging from 28 to 62 years. Of the 13 CFHNs that were observed, eight had greater than five years experience. All the CFHNs were employed in universal services, with 47% fulltime employees and over half (51%) working part-time, one casual CFHN was observed during interactions. All of these CFHNs were registered nurses with specialist qualifications in CFHN either as post registration certificate or post graduate certificate or diploma. Although all 83 CFHNs had recently received family partnership training (Davis & Day 2010), only 40 percent of the CFHNs reported that they had received training in psychosocial assessment and depression screening including the use of the EPDS and domestic violence screening. Mandatory online training was available however, few CFHNs had completed this at the time of data collection for this study.

On average the women who participated in the postnatal component of this study were 30 years of age, ranging from 22–41 years. Of these 20 women, over half (14 out of 20) were born in countries outside of Australia, with 11 born in non-English speaking countries. Of these 20 women, nine were having their first baby. The participants were well educated with 19 of the 20 women having university level qualifications. All of these women were either married or living in *de facto* relationships.

### Data collection

Data collection included observations of interactions between CFHNs and new mothers, brief interviews with the CFHNs following the visit and discussion groups with nurses.

#### Observations

Non-participant observations of interactions between CFHNs and new mothers were conducted at either the home visit conducted by the nurse two to four weeks after birth or in the centre-based visit conducted by the nurse at six weeks after birth. This difference in time points of data collection was unavoidable due to varying approach taken to the implementation of Safe Start policy in each site. Observations occurred between 11 women and seven CFHNs at a home visit and nine observations were conducted at the six week clinic visit with 6 CFHN. The researcher (MR) developed and pilot tested the 4D&4R observation tool [19] and used the tool in a consistent manner, in all settings. The 4D&4R tool [19] was used to orient observations of the dynamics between the women and nurses and support qualitative field notes.

The 4Ds (introDuce, Deliver, Deal and Debrief) were designed to record details about the approach taken by both health professionals (midwives and nurses) to the psychosocial assessment and screening. The 4Rs (React, Respond, Real experience and Reflect) were designed to observed and record detail of the woman’s response, such as how the woman reacted to the asking of sensitive and intimate questions, what were her physical indicators that denote reaction (i.e. flushed face, smiling, frowning etc.); how the woman responded, openness and talkative in her response or did she withdraw from responding using monosyllabic responses or chose to not verbally respond at all; what was the real experience or how congruent did the woman appear (e.g. tearful at discussing traumatic event however denying that she was distressed) and was the woman observed to reflect on the questions being asked (i.e. did she ask to clarify one of the questions or did she raise her response to a previous question at some other point during the interaction) (a more detail discussed on the observation tool is reported in [19].

The observation tool was used in combination with detailed field notes to document verbatim the conversation between the nurse and woman during psychosocial assessment and screening. Notations made within the field notes related to dynamics observed during the interaction between the CFHN and the woman. These interactions were not audio-recorded due to the sensitive nature of the content in these discussions but the field researcher (MR) completed field notes and reflections shortly after observations while drawing on 4D&4R data as a prompt for those notes.

#### Interviews

Brief interviews were conducted with the participating CFHNs directly following the observation. These lasted approximately five to ten minutes obtaining the CFHNs impression of the assessment and if they experienced any challenges or alternatively, if they had felt particularly positive about the style they had used. These data were recorded in field notes and were also not audio-recorded.

#### Discussion groups

Two discussion groups were facilitated from at site B and three discussion groups at site A with CFHNs who conduct psychosocial assessment and depression screening to identify their perceptions and experiences of undertaking psychosocial assessment, beliefs about the nature of the relationship they develop with women/families, training and perceived skills required to undertake psychosocial assessment, their experience of working in multidisciplinary teams and how these services influence outcomes for families (key prompts listed in Table 2). Each group had between 10 to 25 participants, lasting approximately one hour and with the participants’ permission were digitally recorded and transcribed verbatim with all identifying material removed.

**Table 2:** Discussion group questions

	
Discussion group questions CFHNs	
1.	Can you tell us about your experience of conducting Psychosocial assessment and depression screening:
	a. Do you use the SS Q’s?
	b. Do you use the EPDS?
	c. Prompts – can you recall how you felt when you had to ask these questions the first few times?
	d. How do you feel about doing it now?
	e. How do you feel about asking the domestic violence screening questions?
	Are you comfortable with the wording?
	(Maybe) Do you think there could be a different approach to these questions?
2.	How do you think women are prepared for what is entailed in the home visit?
	a. What do you hope to achieve in the first home visit?
3.	What has helped you to incorporate psychosocial assessment and depression screening into your practice?
4.	What challenges have you or your colleagues faced?
5.	What are your views on conducting depression screening with the EPDS? In your experience what have you found to be the best way to use the EPDS?
	a. (Prompt) for example in what part of the interview you would ask the woman to complete the tool. Once the woman has completed the tool and you notice it is high – how do you address this with women
6.	I am also really interest in how you might use your clinical judgment in the process of assessment - Can you describe for me the cues that give you a hunch, about something
	E.g. Strong sense something’s not right, don't get responses or get the opposite what expecting
7.	In what way has your practice changed since you have been incorporating these assessments in your practice?
8.	What, if anything, do you feel has prepared you for working in this way?
9.	What training and support have you been offered and what have you participated in for screening and assessment?
10.	How do you perceive the use of computers will affect this process?
11.	Is there anything else you would like to add?

### Data analysis

The data recorded on the observation tool (4D&4R) was analysed using content analysis [20] and is reported using frequencies. The following questions were used to guide the analysis: how did the CFHN greet the woman, how were the questions introduced, at what point in the consultation was the psychosocial assessment undertaken; on average how much time did the assessment take; how frequently were all psychosocial questions asked; how often were women invited to ask questions, what questions did they ask and how were they framed.

Field note data from the observations and brief interviews with nurses and verbatim transcripts of the discussion group were analysed thematically. The first step in the analysis involved multiple readings and re-readings of the data and listening to the recordings to become immersed in the data [21]. This was followed by identification and labelling of concepts in the data and development of preliminary themes from these concepts. These themes are captured in phrases that where appropriate use the language of the participants. This was an iterative process which involved all researchers discussing the concepts, themes and relationships during the preliminary analysis. Emerging themes and the accompanying data were discussed with the co-authors to ensure reliability of the coding. Concepts and themes were constantly compared with other themes and refined [21]. This process resulted in the identification of four major themes.

## Results

The analysis of observation, interview and focus group data indicated a range of approaches to the psychosocial assessment and depression screening in the postnatal period. The content analysis of the data recorded on the observation tool is presented first, reporting the frequency of assessment and screening. This is followed by a detailed explication of the four major themes that describe and interpret the approach that CFHNs take to assessment and screening. The identified themes are: ‘Engagement: *getting that first bit right’*, ‘Doing some paperwork’, ‘Creating comfort’ and ‘Psychosocial assessment: doing it another way’.

### Frequency of psychosocial assessment and screening

Analysis of data recorded on the observation tool describes how often the nurses conducted the structured assessment in line with the guidelines/policy. Table 3 provides a summary of the frequency of psychosocial assessment and depression screening by nurses in the 20 observed clinical interactions. In all (20 out of 20) of the observed interactions, the CFHN used the EPDS to screen for possible depression, asking the woman to complete the EPDS herself (18 out of 20) and in two situations where the woman’s English was limited; the CFHN read the questions out to the woman.

**Table 3:** Frequency of psychosocial assessment (PSA), Edinburgh Postnatal Depression Scale (EPDS) and Domestic Violence (DV)

**Participant no.**	**Site A: home (11/20 observations)**			**Site B: clinic (9/20 observations)**		
	**PSA safe start**	**EPDS**	**DV**	**PSA safe start**	**EPDS**	**DV**
1		✓				
2		✓	✓			
3		✓				
4		✓				
5		✓				
7				✓	✓	✓
8	✓	✓	✓			
9				✓	✓	✓
11				✓	✓	✓
12				✓	✓	✓
14				✓	✓	✓
15		✓				
17		✓	✓			
18		✓	✓			
21				✓	✓	✓
24		✓				
25					✓	✓
26				✓	✓	✓
30				✓	✓	✓
33				✓	✓	✓

In contrast, only one nurse at site A undertook the structured psychosocial assessment as recommended by the SFE policy, while at site B, the assessment was completed in each of the nine observed interactions. Some nurses at site B used the previous assessment form, as the forms that accompanied the Safe Start policy were still in the ‘roll out’ phase; while others had adopted the Safe Start questions. In six out of the 10 occasions where the psychosocial assessment was conducted, the CFHN asked the woman the questions directly and on four occasions the woman was asked to complete the questions herself. Only one woman sought clarification when the questionnaire was self administered.

At site A, screening for domestic violence was conducted on four occasions and at site B, all the CFHNs undertook domestic violence screening as outlined in the policy. Where the woman’s partner was present in either the home or clinic setting; the CFHN organised private time with the woman so no others were present. In eight out of the 11 home visits observed there was either a partner or others present. On seven occasions the CFHNs did not ask the questions if there was another family member present or at home in the house. However, on one occasion the CFHN was confident that the woman’s partner could not overhear, so she asked the domestic violence questions. In the brief interview following this observation, the CFHN indicated that she had observed some tension between the woman and her partner which she believed may have indicated some interpersonal conflict and control. The following was recorded in field notes (FNW2):

*The CFHN reported that the husband appeared to dominate the conversation that she was trying to have with the woman and was ordering the woman to complete tasks whilst the CFHN was present. The husband had answered for the woman when the CFHN asked routine questions of her, therefore, the CFHN stated that she wanted to explore this relationship dynamic further and the impact this had on the woman*.

In the more formal clinic context (nine out of 20 observations) if others were present, as was the case in three out of nine occasions, the CFHN was able to ask the partner or others to leave the room while the screening was completed. It is also evident that nurses’ comfort with screening for violence influenced practice (nurses’ views on this are addressed below). At site B on the nine occasions when the nurse did assess for domestic violence, these questions immediately followed the psychosocial questions.

### Engagement: *getting that first bit right*

In this study 19 of the 20 observations of nurse-client interactions represented the first encounter between the participating CFHN and the new mother. At the start of the interaction most nurses appeared to prioritise establishing a rapport with the woman by showing respect for the woman and others present, introducing herself, and negotiating where and how the visit should proceed. For example, in one home visit, the CFHN sought permission as to where to ‘set up’ in the following way *‘…hello are you…I’m* (CFHN introduces herself) *from the clinic… where would you like us to go…can we set up my scales here, can we set up on the table?*’ (CFHN3). Encounters in the home appeared to also be influenced by who was present (other children and family members) or whether the baby was feeding, awake or asleep. In this context nurses were observed to be less formal:

CFHN1 - ‘*Hello, how are you? Sorry did we drive your visitors away?’* W1 – *‘They had to go anyway’*Father 1 - ‘*Would you like a cup of tea or something?’* CFHN1- ‘*No thank you, I’m alright.* (Turns to the woman) *We need to do some paperwork and baby check, is she (baby) sleeping? Do you want to do the paper work first?*

In contrast, the clinic environment generated a more formal atmosphere with the prearranged seating directing where the woman and others with her would sit. In this context, CFHN attempted to create a degree of informality by ‘chatting’ with the woman. One nurse for example, started the encounter in the following way: (CFHN11) – ‘*Hi, how you going?*’, the woman responded by asking the CFHN how she is; who then shared her story of the difficulties experienced getting to work that morning ‘*I’ve had one of those mornings…*’ (CFHN11). This appeared to put the family at ease as they laughed and enjoyed the CFHN light heartedness as was noted in field notes (FNW14). In both settings, in this initial effort to build rapport with the woman, CFHNs were also observed to use compliments, particularly about the baby, ‘*she’s so very beautiful isn’t she*’ (CFHN1) or and humour ‘*you’re not ready to give him up yet (laughs)?*’ (CFHN2). These actions were congruent with how nurses talked about the importance of the introduction to the visit in the discussion groups;

*It’s so important you know getting that first bit right. They’re checking you out seeing if you’re good enough and if they can trust you. So it’s really important how you introduce yourself and what needs to be done.* (DG3)

It was also common at this time for the CFHN to introduce what would occur in the overall visit and then to enquire as to how the woman may like to proceed. For example one nurse stated, *‘there are three things we do, the baby check, answer any questions you have and then we do the check up to see how you’re going (looks up to face the woman). What would you like to do first?*’ (CFHN13). The nurse then waited for a response from the woman for guidance as to her preference. Some nurses demonstrated flexibility, by asking the woman ‘*what would you like to do first*’ (CFHN5) or ‘*is bubby asleep? Ok shall I just talk to you then…*?’ (CFHN4).

Less commonly the CFHN appeared rushed or did not appear to take the time needed to establish rapport as evident in this interaction: (Nurse greets the husband at the door) *‘Hello how are you? Is* (Woman’s name) *home’,* (Immediately followed by)*, ‘Have you got somewhere where there’s a bench where I can take the scale? Is it okay to do the baby check?’* (CFHN11).

Although most of the nurses had not met these women before; on several occasions it was observed that the CFHNs had some information about the woman and her family through the referral received from the maternity services. In two instances the CFHN had received information about the woman’s history of mental health problems and domestic violence and raised these issues with the women stating, ‘*It says here that last year you went through a bit of depression, is that true?*’ (CFHN12). Access to or lack of information was also discussed by CFHNs in the groups, ‘*9/10ths of the time you know what you’re going into*’(DG1). However, other CFHNs indicated they were not ‘forewarned’, ‘*sometimes you don’t know what you might find*’ (DG2). When information was provided, the CFHNs tended to feel prepared or ‘*well equipped’* (CFHN11) to undertake the visit and were aware of issues where further exploration may be needed.

### ‘*Doing some paperwork*’

The phrase ‘*doing some paperwork’* was used commonly by nurses when they were providing women with an overview of what would happen in the visit or when they introduced the psychosocial questions and depression screening.

The introduction to and the delivery of the psychosocial assessment and depression screening varied and the participating nurses’ demonstrated capacity to adapt to the context and the needs of the individual woman and family situation. This adaptation occurred despite the fact that nine out of 10 assessments occurred in the more formal clinic context. The assessment questions and screening tools were introduced at varying points in the consultation depending on the woman and the baby. For example, some CFHNs mentioned the assessment and screening, albeit in a ‘roundabout’ way, at the beginning of the visit, ‘*we just need to do some paper work and check her’* (CFHN1) and then at the time of administering the questions a further explanation would be provided ‘*we might do some screening, just a few bits of paperwork…*’ (CFHN13).

In the majority of observations (16 out of 20), the EPDS was introduced by the CFHN prior to administering it. In the other four observations the EPDS was not introduced but rather handed to the woman for her to complete without providing a rationale. A common approach to introducing the EPDS was first to remind the woman that she had completed the same questions at the booking visit in the antenatal period (12 out of 20) *‘…you would have done one of these at the hospital, it’s the Edinburgh Depression Scale’*(CFHN1). However, others simply emphasised*,* ‘…*we routinely do it…we do it on everyone*’ (CFHN1).

The EPDS was most typically introduced at the time in the interaction when the nurse wanted the woman to complete it and on only four out of 16 occasions did a CFHN discuss the rationale for this screening tool during the introduction to the visit stating for example;

*…we firstly screen your baby and we also have a screening tool for you to at this visit. This is to learn a bit more about how you’re feeling so we do check the baby but we want to see how you’re feeling to; is this okay?* (CFHN7).

At times the CFHNs were observed to modify or talked about modifying the questions on the EPDS. This occurred in situations for example, where the woman spoke little English as demonstrated here where the CFHN modifies question 2 of EPDS, ‘*I have looked forward with enjoyment to things*’ to ‘*when you wake first thing in the morning you say, okay I’m going to do this, this and this okay, so let’s do it*’ (CFHN3). On another occasion, the woman was offered a translated version of the EPDS in her first language, Arabic; however, she appeared to have difficulty reading the questions. The woman declined the translated instrument preferring the CFHN to read the questions to her as she explained she was unable to read her first language. In the focus groups, one CFHN explained how she modifies questions regardless of the woman’s ethnicity;

*I’ve been so unhappy, I’ve had trouble sleeping. I say, now divide that up into two questions, not that you’ve had trouble sleeping because the baby’s woken for the feeds. But I always say to them try to focus your answer on the first part of that question…* (DG2)

The CFHNs were also observed to reflect with women on their positive responses on the EPDS and used these questions as prompts for further discussion and exploration;

*This is a really healthy score, but there are a couple of things that you’ve ticked, like not as happy as you usually are…do you think that’s related to the lack of sleep and adjusting to the extra baby?* (CFHN1).

*Blaming yourself unnecessarily you’ve got not very often to that, what sort of things would you blame yourself for?* (CFHN12)

In the discussion groups, the nurses explained this practice ‘*…we always explore, like at the end, when we’ve finished doing it each questions we got a response to, to find out what they mean by that’* (DG2). There were two occasions however, where the CFHN reviewed the EPDS score following the visit while in the car or after the woman had left the clinic setting.

At both sites CFHNs who participated in discussion groups indicated that they were cognisant of when they should refer a woman for additional support or assessment and that they did have access to appropriate referral services for women who had scored positively on the EPDS, ‘*I do know where my role should stop and where to refer to’* (DG2). Various referral sources were described including social work departments within the sites, general practitioners and non-government organisations within their local area and specialist perinatal infant mental health intervention services. The CFHNs were less positive about local referral pathways to mental health services.

### Creating comfort

Most of the CFHNs perceived it was important to ask women the psychosocial questions and to conduct screening for depression and domestic violence ‘…*if you don’t ask you will never know*’ and as a ‘…*opportunity for women to discuss any concerns*, *if they* wish’ (DG1). There appeared to be mixed views on the impact of these questions on the women. Some CFHNs were concerned that the questions may ‘*shock*’ or ‘*surprise*’ women or that the questions may be ‘*confronting*’;

*When you look at your DV [domestic violence] questions or say the Safe Start questions, they're incredibly confronting, whereas EPDS is, I think, is not anywhere near as confronting…* (DG3).

To minimise the impact of the delivery of these questions, CFHNs used a range of strategies, they recognise the importance of creating comfort, allowing time for the woman to feel comfortable and providing practical and emotional support. To create comfort for both the woman and themself, one CFHN described;

*presenting the questions in the most comfortable way as you can in order for them to reflect or give them the opportunity, to say something; because some people have never spoken about it before* (DG2).

The CFHNs also described giving time to this process and allowing women time for discussion ‘*sometimes it takes a whole hour and then she’ll open up*’ (DG2) aids in establishing rapport and creating comfort. At site A, CFHNs described how they ‘*weave them (*the psychosocial assessment questions*) into the conversation so it becomes a part of the conversation*’ (DG3), for example; ‘*do you have practical support with your baby’* is woven into the conversation as ‘… *your partner does your partner help you?*’ (CFHN14) or ‘*Are your family living close by?’* (CFHN10). However, CFHNs described this conversational approach developed with ‘*practice, familiarity and more experience’* (DG3).

Another strategy used by CFHNs to create comfort was providing positive appraisal to women about how they were approaching or caring for their baby. For example, they may deliver forms of encouragement or positive reinforcement ‘*look at you, holding him up close and stroking him, it’s automatic, you’re doing very well’* (CFHN7) ‘*you’re a natural’* (CFHN13). This also included assessing elements of the woman’s emotional state and affect towards the baby as in this example with a woman following disclosure of a recent trauma such as a difficult labour experience ‘*after that experience how was it then, that very early time when she was born, how did you feel about her then?’* (CFHN5).

It was also evident that the CFHNs needed to also create comfort for themselves. Nurses in this study reflected on their own personal life experiences and the impact this may have on the process of asking the psychosocial assessment questions ‘*you might have had some of those experiences yourself and you might not feel comfortable asking a complete stranger about it because it may bring things up*’ (DG2). In discussion groups some CFHNs disclosed that they were uncomfortable with specific questions. For example, some found asking the women about whether they had a history of child sexual abuse was the most challenging question to ask. In the observations with these nurses this question was asked on few occasions where psychosocial assessment was conducted, for example; ‘*I feel a little bit uncomfortable sometimes when you have to stare at a woman and say – have you ever been abused’*(DG3). One nurse noted that it required effort to ask this question ‘*the sexual abuse questions are harder for me to spit out…*’ (DG2). Other CFHNs modified their practice, leaving the sexual abuse in childhood question and only asking it if they received a positive response to one of the domestic violence questions. However, they also suggested that familiarity was one way to overcome this challenge as described by a participating CFHN;

*At first I was so scared when I got a yes to the sexual abuse question; what I’d do with this question… the more times I do it, the more relaxed I am* (DG3).

It appears that the need to create comfort for the woman and for themselves, may have led some CFHNs to adopt a different approach to assessment.

### Psychosocial assessment: doing it another way

During discussion groups and in observing CFHNs practice, it appeared nurses utilise other skills, other than asking recommended, structured questions, to assess women’s social and emotional wellbeing. In the home environment, at the home visit (as illustrated in Table 3), only one nurse conducted the structured psychosocial assessment, only four asked the domestic violence questions but all used the EPDS. During the groups, CFHNs discussed how they assessed women at the home visit in other ways. They emphasised using a range of skills and all of their senses to assess women. This appeared to arise out of the belief that the tools for assessment and screening may be limiting if the only approach used to assessment. As expressed here ‘*they’re tools* (Safe Start questions and EPDS)*…that is all…it’s not a diagnosis*’ (DG1). The subthemes included in this theme ‘Doing it another way’ are: Cloaked in the baby check; gathering information, seeing, hearing, thinking; and having a ‘sixth sense’.

#### ‘Cloaked in the baby check’

CFHNs consider the woman’s perspective is paramount ‘*mum’s come first’* (DG3). They are mindful that ‘*they [the women] are not expecting questions to do with them… their psychosocial… or their depression. They're only focussed on the baby…that’s what they think we’re there for - the baby check.*’ (DG3). CFHNs are aware that they are present in the woman’s home or in the clinic setting ‘*cloaked in the baby check*’ but always in their mind they are aware that they have another agenda; that is the need to undertake a social and emotional assessment of the woman and her family. CFHNs at both sites were observed to be gaining information about the woman’s emotional health and well being in other ways than the structured assessment process.

It appeared the CFHN assessed the woman on how well she was coping with being a new mother by examining the baby’s progress in terms of growth and physical development. For example, this CFHN describes not needing to ask the psychosocial questions because the baby was ‘*Good on the numbers*[baby weight]*… so I had no alarm bells…she’s obviously doing really well*’ (CFHN1). In another example in the interview with the nurse following the observation, the CFHN expressed her concerns to the researcher about the woman based on the assessment of the baby ‘*I am honestly worried about her* [the woman]*; he* [the baby] *is quite small and not attaching at the breast, and she hasn’t been out of the house for 5 weeks – I want to see her again’* (CFHN7).

#### Gathering information - seeing hearing thinking

The nurses’ assessment of the woman and any ‘risk’ to the infant also included observing the home environment. For CFHNs, the appearance of the environment reflected the woman’s level of coping with everyday activities and being a mother. For example, one nurse commented that as she arrived at the house she would observe, ‘*the car parked in the driveway and if there is a* [safety approved] *car seat in*’ (DG2) suggesting she was observing for safety issues. Another nurse talked about her general assessment of the home environment noting whether ‘*…the clothes were like up to the ceiling, I’m thinking this doesn’t look very good… then she [the woman] told me it’s her eighth baby…it’s my washing day today…so there’s nothing wrong with what I saw*’ (DG2).

Nurses were also observed to use individually tailored questions that they believed could ascertain if there were social or emotional issues that needed to be addressed, for example;

*I always ask them if they’re going to have another one (baby) following another one (baby) – it’s my standard question- if they say ‘yes’ then you know they’re doing alright if they say no ‘no more’ then I know there’s something not right* (CFHN3).

#### Intuition

One of the participants described ‘*using all the senses, sounds, smell, everything*’ (DG2) as she considered or took in all aspects about the woman. Others elaborated on this idea talking about the women’s body language ‘…*the moving in the seat, the lack of eye contact at that moment. I suppose you get a sixth sense to maybe probe a little deeper*’ (DG1). The idea of having a ‘*sixth sense*’ and its value in assisting clinical judgement was emphasised by other CFHNs who described how they used their ‘*intuition*’ to assist them in identifying women who needed more time around emotional issues. ‘*It’s not written on their foreheads, maybe she looks happy in front of you and then she starts crying and she’s like a completely different person.. You’re like ah that’s why it didn’t feel right.*’ This was also described as an ‘inkling’ ‘*it’s their body language and how they’re using their words and what their communicating to you, that you get an inkling that something’s not quite right’* (DG3). Some CFHNs described how they altered their practice in order to attend to the woman’s needs once their ‘sixth sense’ had determined that the woman needed some additional support:

*I’ve walked into a house and you can just see that she’s stressed… You say you’ve got a lot on your plate at the moment…let’s just stop right there we’ve got something more important to deal with. We can go back to the baby later* (DG2).

## Discussion

This study examined how CFHNs in NSW undertake psychosocial assessment in the context of the current policy and guidelines requiring both the use of structured tools to determine the psychosocial needs of families following birth, and a flexible partnership approach. Most participating nurses were observed to interact with women and families in a sensitive and caring way and showed a genuine interest in them and their infant. However, the study found varied interpretation of the SFE policy [12] in local practice. Only half of the participating nurses were observed to undertake the structured psychosocial assessment and in all but one case this occurred at site B in the clinic setting suggesting that the service location and/or time point for assessment (2–4 weeks in Site A in the home, Site B at approximately 6 weeks in the clinic) impacts on the way the assessment is delivered. At site A nurses described, both in interviews and in the discussion groups, that they gathered information about a woman, her infant and the family’s needs in other diverse ways. These differing approaches did not apply to the use of the EPDS to screen women for depressive symptoms, which nurses across both sites undertook with ease. It may be that the differences are due to the levels of integration of the new assessment policy rather than the difficulties with the tasks *per se* as screening for depression is a well established practice. Only a few nurses performed domestic violence screening and reflected in the discussion groups their discomfort and difficulties in undertaking this aspect of the assessment. They were similarly uncomfortable asking questions about sexual abuse. Variations in assessment processes have also been observed by Appleton and Cowley [22].

The vast majority of nurses observed in interaction with women demonstrated a genuine regard for women and their families and a capacity to work in partnership [23], even if they may only see the woman and family on this one occasion. Analysis of the observation data showed how nurses want to build rapport and do what Chalmers and Luker [24,25] first described as ‘entry work’ to negotiate the clinical encounter in order to successfully undertake psychosocial assessment [26]. Participants described that their success in gaining entry or not, relied on how they are perceived by families in the initial encounter. Being seen as warm, caring and genuine with a flexible approach, was more likely to facilitate the opportunity to conduct the assessment [8,26-29]. These engagement strategies were observed in the non-verbal and verbal communication of CFHNs such as smiling at the woman, complimenting her on the baby and her mothering and negotiating with her as to how best to commence the visit. This projection of optimism by the nurse is described as an initial phase of interaction and was also observed in public health nurses in Canada interacting with women [28]. The nurses also conveyed a happy and friendly disposition at the onset of contact with the woman sending a message of the nurse being easy going and that the rest of the assessment may consequently go well. This also assisted in establishing initial rapport with the women [28]. This sensitivity to the commencement of the visit establishes a sense of attunement to the woman which Oberle and Tenove [30] describe as a discrete balance between the nurse and the woman. Over time this sensitivity to women assists the CFHN to attune to their strengths, capacities and vulnerability or potential risk [30].

However, it is evident that nurses experienced a dissonance or tension, being cognisant of the need to work in partnership with women and families, demonstrating a high level of respect and genuineness [23] for families, while at the same time responding to the mandate to assess for risk in a structured, population-based approach. Some nurses were also aware that this tension or confusion may also be experienced by families who believe that the nurse is focused on ‘checking’ the baby and then subsequently discover the nurse is also monitoring the progress of the mother by asking sensitive and intimate questions to determine the mental health and well being of the woman [31]. Nurses in this study guarded against this by ‘cloaking’ the assessment in the baby check and downplaying the assessment as ‘doing some paperwork’.

One of the central findings of the study is the way in which many of the nurses at both sites were observed and reported taking a more flexible approach to assessment. To gather a ‘full picture’ about a woman and family, the CFHNs described using a range of skills to assess a woman’s needs which at site B were in addition to the structured questions and at site A were instead of these questions. For example, participants used the assessment of the baby and the mother-baby interaction as a way to ‘tap’ into a mother’s well being and how she is managing in her role as a mother of a new baby. The notion of doing the psychosocial assessment in another way has also been reported by Armstrong and Small [32] who found that maternal and child health nurses relied on their own assessments which often overrode protocol. The complex interactive occurrence of other processes occurring at the same time as routine assessment such as clinical judgement can be supported in this context and is under reported [33].

Nurses used observation skills to take in the environment as well as intuition where they drew on extensive clinical experience to carefully attune to the non verbal messages given by the woman and others present. This was captured by one nurse in the phrase, ‘*seeing hearing thinking*’. Wilson [34] describes this as a comprehensive form of ‘health surveillance’ but cautions along with others [31,35] that what is mostly considered a routine and unproblematic aspect of CFHN practice, may in fact be interpreted by women and families as a form of State control and regulation. This level of surveillance can occur in both the home and the clinic setting but it is largely unknown what impact this has on women [36].

In an attempt to reduce the impact of surveillance Jack et al. [31] describes that health professionals must work at assisting families to overcome fear, enabling a trust relationship to develop and to identify mutual or common ground. However, the other response that may occur is over compliance on the part of the woman to accommodate the nurse and to seek a relationship with the nurse [34]. In this study, nurses acted to both establish a relationship but also conceal the full purpose of the visit. Both the ‘engagement work’ of the nurse and the engagement by women and families raise ethical issues about the actual intent of the service, what families are seeking and most importantly, how this is achieved and how boundaries are established in the developing relationship [37]. This was echoed by Kardamanidis [27] who found in interviews with CFHNs providing sustained nurse home visiting, that women were more likely to disclose sensitive information when time was taken to build a trusting relationship than if simply asked a series of prescribed questions. Although there was some evidence of adaptation of psychosocial assessment processes in response to women with limited English, for example; modification of EPDS, the issues of conducting psychosocial assessment across cultures and building relationships with women who require language support, however, remains unresolved.

Some studies [32,38] suggest that women prefer to talk with a professional about their concerns or worries rather than complete a questionnaire. Similarly nurses also appear to prefer to talk with women and to make assessments about their mental health and well-being by using clinical judgement informed by gathering information in a range of ways, one of which might include a screening tool. Nurses in this study chose to adopt a more relational approach to assessment where they monitor for social cues and recognise the importance of building a relationship with families and that it is on the basis of this relationship that they are able to effectively assess the needs of women. This practice is consistent with that recommended by Cowley and Houston [39] who reject a structured format believing that it does not allow for the flexibility required to elicit sensitive information and suggest that these issues should be uncovered by the health professional during their ongoing contact with a woman. Nurses described observing the non verbal and verbal cues given by women and that they effectively ‘monitor’ women’s response in order to manage any distress [40].

Overall, nurses found this work challenging and described in the discussion groups that there were aspects of the assessment that they felt uncomfortable with for example screening for domestic violence and asking about child abuse. Other researchers [39,41] have raised concerns about the level of skill and the approach used by health professionals when conducting assessments related to obtaining sensitive information. Many CFHNs in this study felt they are inadequately prepared to undertake psychosocial assessment and have limited skills in eliciting and responding to sensitive information and the needs of women and families [40,42]. To manage CFHNs discomfort whilst introducing assessment, in this study CFHNs reported ‘cloaking’ psychosocial assessment in the baby check and ‘doing some paperwork’. These findings suggest that services need to invest in ongoing training, support and supervision for CFHNs to improve their skills and confidence in relating the purpose of assessment to families.

### Study limitations

This study is based on small numbers of observations but provides a richness of qualitative data that may not be generalised, but the commonality of themes expressed by participants suggests similarity in the experience [43]. The sample included experienced CFHN, who may have felt more confident to participate in the research, and women who have received a university level of education. The sample thus may not reflect the general population of women using the service or CFHN providing the service. This may have impacted on the interaction. This study was also conducted across two sites that differed in both the location where assessment was conducted. Home versus clinic and at two different points in time, 2 weeks versus 6 weeks following birth. Some of the differences in interaction that were observed may have therefore occurred because the infant was 2 weeks versus 6 weeks old or because of the location.

## Conclusion

This study explored the approach that nurses take to psychosocial assessment and depression screening in the context of the current debates and policy that requires the use of structured tools and assessments together with a flexible, partnership approach to working with families. The nurses managed this tension in two ways, first by doing the paperwork and second, in some instances by doing the assessment in an unstructured way using their observational skills and intuition to inform a clinical judgement. Whilst the use of structured assessment together with a partnership approach is consistent with best practice [44-46], nurses report it is challenging to work this way.

## Abbreviations

CFHN: Child and family health nurse; SFE: Supporting families early package; NSW: New south wales; MR: Mellanie rollans; DG: Discussion groups; W: Woman; FN: Field note; PSA: Psychosocial assessment; EPDS: Edinburgh postnatal depression scale; DV: Domestic violence.

## Competing interests

The authors declare that they have no competing interests.

## Authors’ contributions

MR: Carried out the data collection, participated in the data analysis and the drafting of this manuscript. VS: Assisted in the design of the study, participated in the data analysis and the drafting of this manuscript. LK: Assisted in the design of the study, participated in the data analysis and the drafting of this manuscript. TC: Assisted in the design of the study, participated in the data analysis and the drafting of this manuscript. All authors read and approved the final manuscript.

## Authors’ information

Mellanie Rollans is currently a PhD Candidate in the School of Nursing and Midwifery at the University of Western Sydney. Her background is in mental health nursing and psychology, with a keen interest in research, early intervention and preventative strategies to enhance social and emotional wellbeing.

Virginia Schmied is Professor (maternal infant and family health) in the School of Nursing and Midwifery at the University of Western Sydney. She is a midwife and nurse who facilitate a program of research related to perinatal mental health, transition to parenthood and infant feeding decisions.

Lynn Kemp is Associate Professor and Director of the Centre for Health Equity Training Research and Evaluation, part of the Centre for Primary Health Care and Equity, University of New South Wales. Originally trained as a nurse, she received her doctorate from the University of Western Sydney. Her research interests focus on interventions to improve outcomes for vulnerable families and communities, with a particular interest in early childhood intervention, and community, health system and policy interventions to improve health equity.

Tanya Meade Associate Professor with the School of Social Sciences and Psychology at the University of Western Sydney and a research fellow with the School of Medicine, University of Sydney. Her research and teaching is in the area of clinical health psychology with a focus on co morbidities, assessment and psychological adjustment.

## Pre-publication history

The pre-publication history for this paper can be accessed here:

http://www.biomedcentral.com/1472-6963/13/133/prepub
